# Coordination Dynamics and Coordination Mechanism of a New Type of Anticoagulant Diethyl Citrate with Ca^2+^ Ions

**DOI:** 10.1155/2013/354736

**Published:** 2013-12-24

**Authors:** Jin Han, Jun-Fa Xue, Meng Xu, Bao-Song Gui, Li Kuang, Jian-Ming Ouyang

**Affiliations:** ^1^Department of Nephrology, The Second Hospital of Xi'an Jiaotong University, Xi'an 710004, China; ^2^Institute of Biomineralization and Lithiasis Research, Jinan University, Guangzhou 510632, China

## Abstract

Diethyl citrate (Et_2_Cit) is a new potential anticoagulant. The coordination dynamics and coordination mechanism of Et_2_Cit with Ca^2+^ ions and the effect of pH on the complex were examined. The result was compared with that for the conventional anticoagulant sodium citrate (Na_3_Cit). The reaction order (*n*) of Et_2_Cit and Na_3_Cit with Ca^2+^ was 2.46 and 2.44, respectively. The reaction rate constant (*k*) was 120 and 289 L*·*mol^−1^ *·*s^−1^. The reverse reaction rate constant (*k*_re_) was 0.52 and 0.15 L*·*mol^−1^ *·*s^−1^, respectively. It is indicated that the coordination ability of Et_2_Cit with Ca^2+^ was weaker than that of Na_3_Cit. However, the dissociation rate of the calcium complex of Et_2_Cit was faster than that of Na_3_Cit. Increased pH accelerated the dissociation rate of the complex and improved its anticoagulant effect. The Et_2_Cit complex with calcium was synthesized and characterized by elemental analysis, XRD, FT-IR, ^1^H NMR, and ICP. These characteristics indicated that O in –COOH and C–O–C of Et_2_Cit was coordinated with Ca^2+^ in a bidentate manner with 1 : 1 coordination proportion; that is, complex CaEt_2_Cit was formed. Given that CaEt_2_Cit released Ca^2+^ more easily than Na_3_Cit, a calcium solution was not needed in intravenous infusions using Et_2_Cit as anticoagulant unlike using Na_3_Cit. Consequently, hypocalcemia and hypercalcemia were avoided.

## 1. Introduction

An anticoagulant must be added to dialysates to prevent blood solidification *in vitro* (in a dialysis machine). Sodium citrate (Na_3_Cit) is an important anticoagulant used in clinical settings [1–3]. However, using Na_3_Cit as an anticoagulant easily causes hypocalcemia and hypercalcemia [4, 5] because of the strong chelating ability of Na_3_Cit with Ca^2+^ ions. Given this ability, the dissociation metabolism of the formed chelate CaCit *in vivo* takes 30 min. Using Na_3_Cit also negatively affects the maintenance of coagulation stability of high-risk hemorrhage patients *in vivo*, which easily causes complications such as hypocalcemia during or after dialysis.

Our group has previously synthesized a new anticoagulant [6], namely, diethyl citrate (Et_2_Cit). The anticoagulant mechanism of Et_2_Cit is based on the formation of Ca^2+^ with Et_2_Cit. This formation decreases the Ca^2+^ concentration in blood and inhibits prothrombin conversion into thrombin, thereby influencing the anticoagulant effect. The large steric effect of Et_2_Cit weakens the coordination of Ca^2+^ ion compared with that of Na_3_Cit. Therefore, hypocalcemia and hypercalcemia can be avoided using Et_2_Cit as anticoagulant [7]. The frequency of blood gas analyses can also be lessened by repeatedly taking the venous blood of patients to monitor serum calcium levels, which can help relieve the pain of patients and the workload of nurses.

The stability of the complex of Et_2_Cit with Ca^2+^ (CaEt_2_Cit) is reportedly weaker than that of CaCit [8]. At pH 7.4 and 37°C, the stability constants (*K*_*s*_'s) are 1988 for CaCit and 231 for CaEt_2_Cit. However, several problems remain unsolved when Et_2_Cit is used as an anticoagulant. These problems include the reaction kinetics of Et_2_Cit with Ca^2+^ and coordination reaction mechanisms, as well as the composition and characterization of the complex. Accordingly, the coordination dynamics of Et_2_Cit and Na_3_Cit with Ca^2+^, as well as the influencing factors, were studied. The underlying coordination principle was also proposed.

## 2. Materials and Methods

### 2.1. Instruments and Reagents

The instruments used were as follows: CHN–O– rapid type element analyzer (Foss-Heraeus Company), Bruker AM 500 nuclear magnetic resonance (NMR) spectrometer (with CDCl_3_ as solvent and TMS as internal standard), Nicolet-170 SX type FT-IR spectrometer, *D*/max 2400 (Rigaku) X-ray diffractometer, inductively coupled plasma emission spectrometry (ICP) system (PE Company, USA), PHS-3C pH meter (Shanghai Precision & Scientific Instrument Co., Ltd., China), and sodium chloride injection system (Wuhan Binhu Double-Crane Pharmaceutical Co., Ltd., China).

All chemical reagents used were of analytical grade. Et_2_Cit was prepared in our laboratory (99.3% purity) [6].

### 2.2. Experimental Methods

#### 2.2.1. Reaction Rate Constants of Et_**2**_Cit and Na_**3**_Cit with Ca^2+^

CaCl_2_ and Et_2_Cit solutions (2.0 mmol/L) were prepared and mixed. A calcium-ion-selective electrode was used to determine the change in electrode potential of the mixed solution with reaction time at pH 7.4 and 37°C under stirring. The result was then compared with that of Na_3_Cit.

The linear regression equation of the calcium ion-selective electrode was *y* = 29*x* + 69 (where *y* is the electrode potential and *x* is –p(Ca^2+^). The concentration of Ca^2+^ [*c*(Ca^2+^)] at *t* time was also calculated. Given that CaCl_2_ was mixed with Na_3_Cit or Et_2_Cit (1 : 1) and that the reaction of Ca^2+^ with Na_3_Cit or Et_2_Cit was equal in solution [7], the following reaction rate equation can be established using *r* to represent the reaction rate:

(1) $$\begin{array}{c} r=\;kc^{n}, \end{array}$$

where *k* is the reaction rate constant and *n* is the reaction order. Assuming that *x* is the amount of Ca^2+^ substance concentration that disappeared at *t* time, that is, *x* = *a* − *c* (Ca^2+^), the following can be obtained by arranging formula (1):

(2) $$\begin{array}{c} r=-\frac{dc}{dt}=-\frac{d\left(a-x\right)}{dt}=\frac{dx}{dt}=k{\left(a-x\right)}^{n}. \end{array}$$

After logarithm on both sides we get

(3) log⁡⁡r⁡=log⁡⁡(−d(a−x)dt)=log⁡⁡k+nlog⁡⁡(a−x)=log⁡⁡k+nlog⁡⁡c.

From the plot of *x*  versus *t*, we can calculate the tangent slope of the curve *dx*/*dt*, which is the reaction rate of various points. Formula (3) shows a linear relationship between log⁡⁡*r* and log⁡⁡*c*(Ca^2+^). In the diagram of log⁡⁡*r* on log⁡⁡*c*(Ca^2+^), the slope of the straight line is the reaction order *n*, whereas the intercept is log⁡⁡*k*.

#### 2.2.2. Effect of pH on Reaction Rate

The pH of the system was adjusted to 6.0, 7.4, and 8.0. Then, the effect of pH on *k* and *n* was determined.

#### 2.2.3. Synthesis of Diethyl Citrate Calcium Complex Crystal

About 1.665 g (15 mmol) of anhydrous CaCl_2_ was completely dissolved in water. Then, 1.241 g (5 mmol) of Et_2_Cit was slowly trickled under stirring. The pH was adjusted to 7.0 after obtaining a colorless and transparent solution. The solution was sealed with a plastic wrap having holes and then placed in an oven at 37°C for slow volatilization and crystallization. The precipitated colorless, needle-like crystals were filtered, washed with anhydrous ethanol, dried, and characterized. The methods of characterization included elemental analysis, X-ray powder diffraction (XRD), Fourier-transform infrared spectroscopy (FT-IR), ^1^H NMR, and ICP.

## 3. Results and Discussion

### 3.1. Reaction Rate Equation of Et_**2**_Cit and Na_**3**_Cit with Ca^2+^

**Figure 1:**
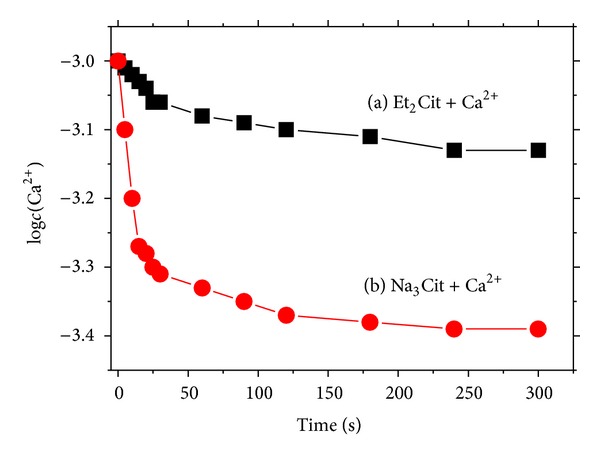
Changes in Ca^2+^ concentration with reaction time in different systems: (a) Et_2_Cit-Ca^2+^ system and (b) Na_3_Cit-Ca^2+^ system.

The change in concentration of free Ca^2+^ ion [*c*(Ca^2+^)] with *t* in reaction system of Et_2_Cit and Na_3_Cit with CaCl_2_ is shown in Figure 1. A rapid decrease in *c*(Ca^2+^) was observed with prolonged *t* from 0 s to 30 s. This finding indicated that Et_2_Cit or Na_3_Cit was rapidly coordinated with Ca^2+^. At *t* = 30 s, *c*(Ca^2+^) decreased from 1.0 mmol/L to 0.49 mmol/L in the Na_3_Cit system and from 1.0 mmol/L to 0.87 mmol/L in the Et_2_Cit system. *c*(Ca^2+^) slowly decreased when *t* > 120 s, indicating that the system was in a dynamic equilibrium of complexation dissociation.

**Figure 2:**
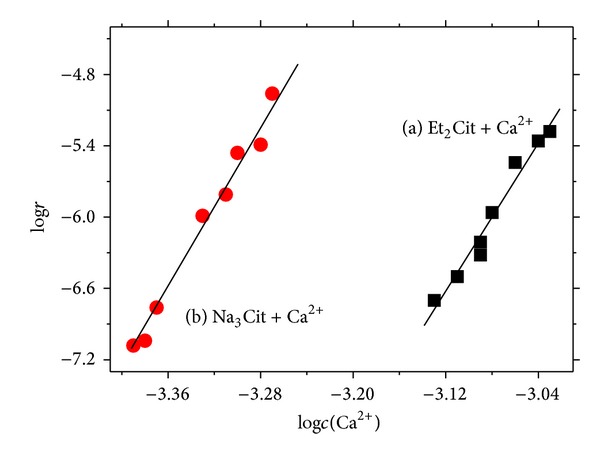
Plots of log⁡⁡*r*-log⁡⁡*c*(Ca^2+^) in different systems: (a) Et_2_Cit-Ca^2+^ system and (b) Na_3_Cit-Ca^2+^ system.

The tangent slope (*dx*/*dt*) of points on the curve, that is, the reaction rate *r* of each point formula (2), can be obtained according to Figure 1. In the diagram of log⁡⁡*r*  versus  log⁡⁡*c*(Ca^2+^) (Figure 2), the slope of the line was the reaction order *n* (formula (3)). The intercept of the line was log⁡⁡*k* in Figure 2, as shown in the following:

(4) $$\begin{array}{c} {\text{Et}}_{2}\text{Cit-Ca}\;\text{system}:n=2.46;\;\;\log k=2.06,\;\;\text{so}\;k=120;\\ {\text{Na}}_{3}\text{Cit-Ca}\;\text{system}:n=2.44;\;\;\log k=2.46,\;\;\text{so}\;k=289. \end{array}$$

The reaction rate equations of Et_2_Cit and Na_3_Cit with Ca^2+^ were as follows:

(5) $$\begin{array}{c} {\text{Et}}_{2}\text{Cit-Ca}\;\text{system}:r=\;ka^{2.46}=120a^{2.46},\\ {\text{Na}}_{3}\text{Cit-Ca}\;\text{system}:r=\;ka^{2.44}=289a^{2.44}. \end{array}$$

Given that *k* can directly reflect the reaction rate, (5) shown that the complexation rate of Na_3_Cit with Ca^2+^ was faster than that of Et_2_Cit.

The anticoagulant mechanism of Na_3_Cit and Et_2_Cit was based on the combination of calcium ion (Ca^2+^) in serum, as well as the reduced concentration of free Ca^2+^ in plasma that disturbed the blood clotting process from reaching the anticoagulation effect *in vitro* [9–11]. However, the strong coordination ability of Na_3_Cit, particularly as an anticoagulant, can coordinate a large number of Ca^2+^ ions in the blood. This phenomenon can lead to the low serum concentration of calcium in patients, as well as to hypocalcemia and all kinds of complications [12–15]. Therefore, calcium is needed to be replenished in the anticoagulation process of Na_3_Cit [16]. Meanwhile, calcium citrate [CaCit] can dissociate during the metabolism and release Ca^2+^ after entering the body in the dialysis process. Additionally, hypercalcemia easily ensued in patients with presupplementary Ca^2+^. Therefore, the incidence of hypocalcemia and hypercalcemia can be reduced if we can reduce the coordination ability of anticoagulant.

The reaction rate was equal to the inverse reaction rate when the reaction reached equilibrium, as shown in the following:

(6) $$\begin{array}{c} k{\left(a-x\right)}^{n}=k_{\text{re}}x^{n}. \end{array}$$

The above equation can be written as follows [17]:

(7) $$\begin{array}{c} \frac{x^{n}}{{\left(a-x\right)}^{n}}=\frac{k}{k_{\text{re}}}=K_{s}, \end{array}$$

where *k* is the reaction rate constant, *k*_re_ is the inverse reaction rate constant, and *K*_*s*_ is the complex stability constant.

In a previous article [8], the *K*_*s*_ values of CaEt_2_Cit and CaCit were 231 and 1988 at pH 7.4 and 37°C, respectively, and the *k* values in the coordination reaction of Et_2_Cit and Na_3_Cit with Ca^2+^ were 120 and 289 L·mol^−1^·s^−1^, respectively. According to (7), *k*_re_ of Et_2_Cit and Na_3_Cit with Ca^2+^ in the coordination reaction were 0.52 and 0.15 L·mol^−1^·s^−1^, respectively. Thus, the rate of decomposition and release of Ca^2+^ was faster for CaEt_2_Cit than for CaCit. The above results indicated that Et_2_Cit can complex with Ca^2+^ and reduce the free Ca^2+^ concentration during anticoagulation; thus, anticoagulation can be achieved. Meanwhile, the complexing ability of Et_2_Cit with Ca^2+^ was weaker than that of Na_3_Cit. After Et_2_Cit coordinated with Ca^2+^, the Ca^2+^ releasing rate of CaEt_2_Cit was faster than that of CaCit. Therefore, the occurrence of hypocalcemia in patients can be avoided. Moreover, only a small amount of calcium or none at all was needed using Et_2_Cit as anticoagulant during dialysis unlike using Na_3_Cit. Thus, the occurrence of hypercalcemia can be avoided using Et_2_Cit as an anticoagulant.

### 3.2. Effect of pH on Reaction Rate

At present, the main dialysates in clinical practice are bicarbonate and acetic dialysis liquid. The pH of acetate dialysate is generally controlled to remain at 6.0 to 7.2 [18]. In [19], the pH range of the dialysate is 5.3–8.2. At the entrance of the dialysis machine, the pH of a patient's whole blood was between 7.15 and 7.4, whereas the pH of the exports of the dialysis machine was between 6.2 and 7.4.

In the dialysis process, the pH values of different dialysates varied. The acidities of different anticoagulants also differed. Therefore, the pH of blood in the dialysis process also changed. Considering that Na_3_Cit was a strong base-weak acid salt, 1 mol of Na_3_Cit contained 3 mol of carboxylate (COO^−^), wherein Na_3_Cit was alkaline. Therefore, when Na_3_Cit was used as an anticoagulant, the blood pH decreased and metabolic alkalosis likely ensued.

**Figure 3:**
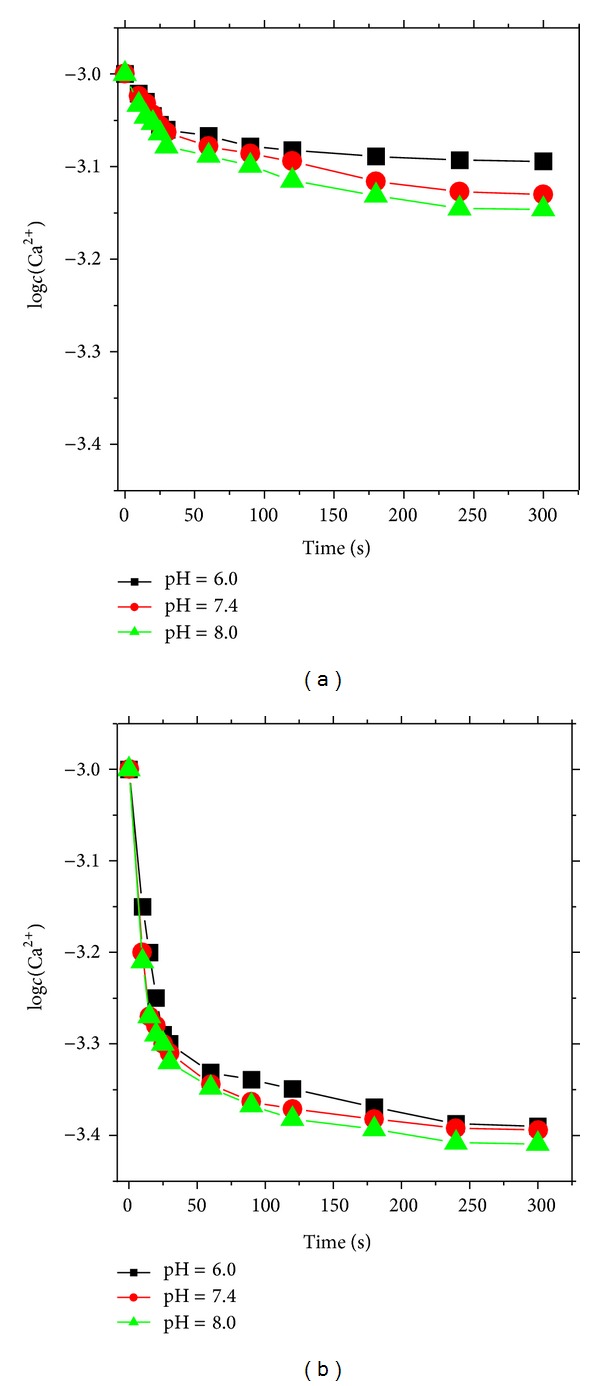
Plots of concentration change of free Ca^2+^ ions with reaction time under different pH conditions: (a) Et_2_Cit and (b) Na_3_Cit.

Considering that one Et_2_Cit molecule only had one –COO^−^, the possibility of causing alkalosis was significantly reduced when Et_2_Cit was used as anticoagulant. With increased pH from 6.0 to 8.0, free *c*(Ca^2+^) decreased faster in the system (Figure 3) because increased pH benefited the ionization of –OH and –COOH of Et_2_Cit or Na_3_Cit, which in turn benefited the coordination with Ca^2+^.

Table 1 shows the reaction rate constants *k* of Et_2_Cit and Na_3_Cit with CaCl_2_, as well as the complex dissociation rate *k*_re_ when the pH values of the system were 6.0, 7.4, and 8.0. The reaction rate and dissociation rate of the complex were found to accelerate with increased pH. The reaction rate of Et_2_Cit and Na_3_Cit with CaCl_2_ was influenced by pH because H^+^ inhibits the ionization of the active H of –COOH in Et_2_Cit molecule, as well as changing the course of coordination reaction. Thus, the reaction rate constant and reaction order changed.

Within pH 6.0–8.0, the pH increase accelerated the dissociation rate of the complex. With increased pH from 6.0 to 8.0, *k*_re_ of the Et_2_Cit-CaCl_2_ system increased from 0.04 to 19.8, whereas *k*_re_ of the Na_3_Cit-CaCl_2_ system increased from 0.03 to 6.79. The dissociation rate of the complex for the coordination of Et_2_Cit and Na_3_Cit with calcium under an alkaline condition was faster than that under an acidic condition. Therefore, the pH increase of anticoagulants such as Et_2_Cit and Na_3_Cit and dialysis under alkaline conditions achieved the purpose of anticoagulation and avoided the occurrence of dialysis acidosis, thereby improving the survival rate and quality of life.

### 3.3. Research on Et_**2**_Cit and Ca Complexes

#### 3.3.1. Elemental Analysis and Ca Content as Determined by ICP

To further study the coordination of Et_2_Cit with Ca^2+^, the complex of Et_2_Cit with Ca^2+^ was synthesized. Its composition was analyzed using elemental analysis and ICP, and the results are shown in Table 2. Et_2_Cit was found to form the complex of CaEt_2_Cit with Ca^2+^ in 1 : 1 coordination ratio. Therefore, the experimental value was consistent with the theoretical value.

#### 3.3.2. XRD Analysis

**Figure 4:**
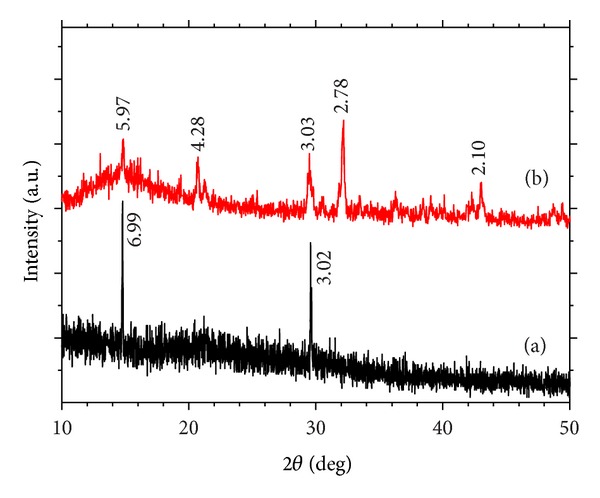
XRD patterns of CaEt_2_Cit and CaCl_2_: (a) CaEt_2_Cit and (b) CaCl_2_.

Figure 4 is the XRD pattern of CaCl_2_ and CaEt_2_Cit crystals. The diffraction peaks of CaCl_2_ appeared at *d* = 5.97, 2.78, 3.03, 4.28, and 2.90 Å (Figure 4(a)), whereas the diffraction peaks of the complex appeared at *d* = 6.99 and 3.02 Å.

#### 3.3.3. FT-IR Analysis

**Figure 5:**
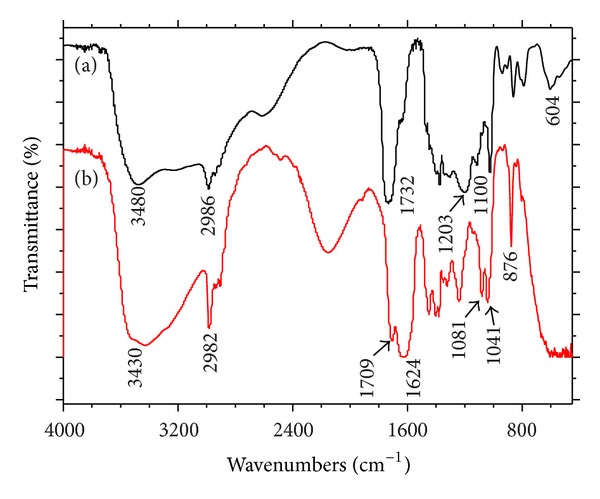
FT-IR spectra of Et_2_Cit and its complex CaEt_2_Cit: (a) Et_2_Cit and (b) CaEt_2_Cit.

The FT-IR spectra of Et_2_Cit and CaEt_2_Cit complex are shown in Figure 5. The wavenumbers of the main absorption peaks are shown in Table 3 [20].

1. The peak at 3430 cm^−1^ was due to the stretching vibration of the hydroxyl group in the CaEt_2_Cit complex, which red shifted by approximately 50 cm^−1^ more than that of Et_2_Cit (3480 cm^−1^), indicating a hydrogen bond.
2. The carbonyl absorption peak (C=O) of CaEt_2_Cit split into two peaks, which were 1709 and 1624 cm^−1^, respectively, indicating two different coordination environments in carbonyl. The position of both peaks red-shifted by approximately more than 30 and 110 cm^−1^ compared with the carbonyl absorption peaks of Et_2_Cit at 1736 cm^−1^. This finding indicated that the carbonyl of Et_2_Cit was coordinated with the calcium ions and was consistent with the change in the carbonyl characteristic absorption peak before and after coordination, as reported in [20].
3. The absorption peak of the symmetric stretching vibrations of (C–O–C) in C–O–C of Et_2_Cit was at 1100 cm^−1^. However, the peak split into two in the complex, that is, at 1081 and 1041 cm^−1^, respectively. This phenomenon was ascribed to one of the three C–O–C groups of the Et_2_Cit molecular complex with Ca^2+^, in which C–O–C absorption was bimodal and red shifted.
4. The peak at 2982 cm^−1^ was the absorption peak of the methyl hydrocarbon of CaEt_2_Cit. It did not significantly change compared with the absorption peak of Et_2_Cit methyl hydrocarbon (2986 cm^−1^).

#### 3.3.4. ^1^H NMR 

**Figure 6:**
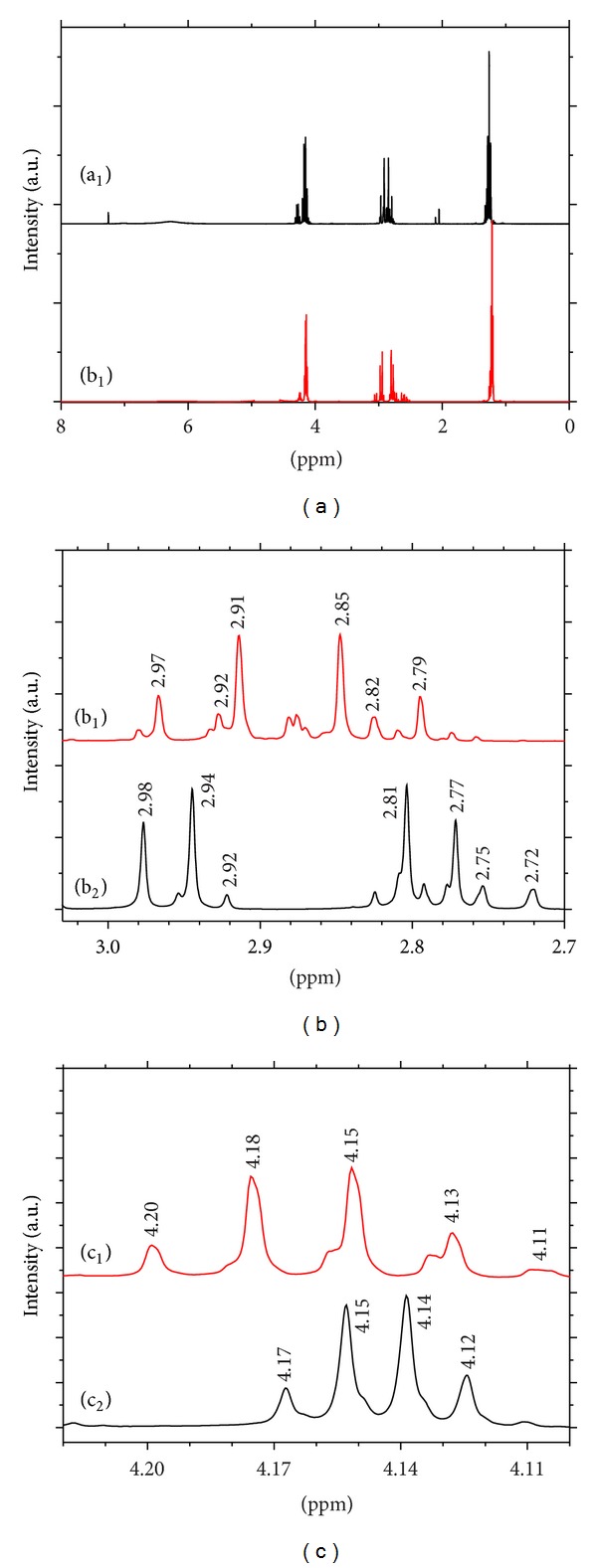
^1^H NMR spectra of Et_2_Cit and its complex CaEt_2_Cit. (a_1_, b_1_, and c_1_) are Et_2_Cit; (a_2_, b_2_, and c_2_) are CaEt_2_Cit. (a) Total spectra, (b) *δ* = 2.70–3.00 section, (c) *δ* = 4.10–4.21 section.

The ^1^H NMR spectra of Et_2_Cit and CaEt_2_Cit were studied using CDCl_3_ as a solvent, and the results are shown in Figure 6. The absorption peaks of ^1^H NMR are shown in Table 4.

1. The proton peaks of the ligand at *δ* = 7.26 and 6.28 ppm disappeared, indicating that –COOH participated in the coordination reaction. Meanwhile, the hydrogen in –OH group is very active; it can be easily dissociated and be partially or entirely substituted by deuterium in CDCl_3_ solution.
2. At 2.70 ppm to 3.0 ppm, the two groups of Et_2_Cit quartets were –CH_2_C=O (Figure 6(b)). –CH_2_C=O groups occurred in different chemical environments, that is, 1,3-Et_2_Cit and 1,5-Et_2_Cit. The physical and chemical properties of the two isomers were very similar, so the two peaks did not significantly differ. After CaEt_2_Cit was generated, the chemical environment of Et_2_Cit changed and resulted in obvious dispersion and specificity of the two peaks of 2.70 ppm from 3.0 ppm. This result indicated that after 1,3-Et_2_Cit and 1,5-Et_2_Cit coordinated with calcium ions, the property difference of the two formed complexes increased compared with those of the original two ligands.
3. At 2.70 ppm to 3.0 ppm, the H peaks of –CH_2_– shifted from 2.85 ppm to 2.91 ppm, and then to 2.81 ppm to 2.94 ppm after Et_2_Cit coordinated with calcium. This finding was due to the O in –OCH_2_ that coordinated with Ca, consistent with the IR spectra.
4. The peak at *δ* = 4.0 ppm was assigned to –OCH_2_ of –COOCH_2_CH_3_ (Figure 6(c)). Compared with Et_2_Cit (*δ* = 4.13 ppm to  4.20 ppm), this peak of the complex (*δ* = 4.12 ppm to  4.17 ppm) shifted to a high field. This finding indicated the weakening of the induction effect of attracting electrons of O in –OCH_2_ from H after the O atom in –OCH_2_ coordinated with Ca. Thus, the total electron density of H increased, and the absorption peaks moved to a high field.

Elemental analysis, ICP, XRD, FT-IR, and ^1^H NMR revealed that Et_2_Cit formed a 1 : 1 complex with Ca^2+^, that is, CaEt2Cit.

#### 3.3.5. Coordination Mechanism

**Figure 7:**
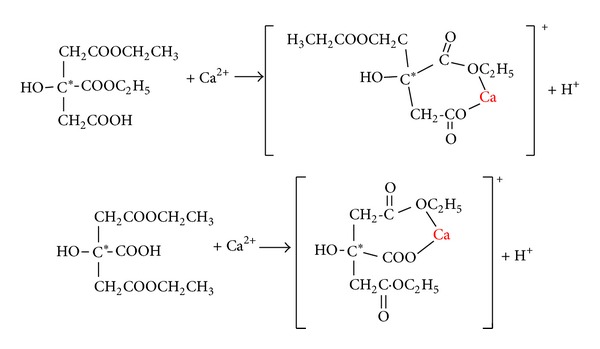
Schematic of the coordination of Ca^2+^ ion with two isomers of anticoagulant Et_2_Cit. The asterisk ∗ shows the center C atom of Et_2_Cit.

The above results showed that Ca^2+^ was coordinated with Et_2_Cit. O in –COO and C–O–C of Et_2_Cit was coordinated with Ca^2+^ in bidentate ligand. Two kinds of –OCH_2_CH_3_ had different chemical environments in the crystals, that is, 1,3-CaEt_2_Cit and 1,5-CaEt_2_Cit. However, their proportions were still difficult to ascertain because of their similar physical and chemical properties. Based on the above characterization results, two kinds of coordination of Et_2_Cit with Ca^2+^ are shown in Figure 7.

We rule out the possible coordination of hydroxyl group of Et_2_Cit based on the reason that the FT-IR (Figure 5) and ^1^H NMR spectra (Figure 6) have confirmed that one of the carbonyl of Et_2_Cit was coordinated with the calcium ion. When one –COOH and one –COOCH_2_CH_3_ in Et_2_Cit were coordinated with calcium ion, the –OH group and the coordinated Ca ion were separated on opposite sides of the center C atom of Et_2_Cit (Figure 7); thus –OH cannot coordinate with calcium ion because of the space steric hindrance.

## 4. Conclusion

The coordination dynamics and effect of Et_2_Cit and Na_3_Cit pH on Ca^2+^ in saline water were studied. In 37°C saline water, the coordination dynamics equations of Et_2_Cit and Na_3_Cit with Ca^2+^ were *r* = 120*a* ^2.46^ and *r* = 289*a* ^2.44^, respectively. The reverse reaction rate constants (*k*_re_'s) of coordination with CaCl_2_ were 0.52 and 0.15 L·mol^−1^·s^−1^ for Et_2_Cit and Na_3_Cit, respectively. The dissociation rate of Ca^2+^ of CaEt_2_Cit was faster than that of CaCit. The increased pH accelerated the dissociation of the complex. With increased pH from 6.0 to 8.0, *k*_re_ of Et_2_Cit-CaCl_2_ increased from 0.04 to 19.80, which was beneficial in improving the anticoagulant effect. Et_2_Cit and Ca^2+^ were coordinated to form a 1 : 1 complex, and O atoms in –COOH and C–O–C of Et_2_Cit were coordinated with Ca^2+^ in bidentate ligand. Et_2_Cit was able to coordinate with Ca^2+^, and its release capacity of Ca^2+^ was stronger than that of Et_2_Cit. Thus, it did not require an intravenous infusion of calcium when used as an anticoagulant, thereby avoiding hypocalcemia and hypercalcemia that can be caused by Na_3_Cit. Overall, Et_2_Cit was a better anticoagulant than Na_3_Cit.

## Figures and Tables

**Table 1:** Reaction rate constant and reaction order of Et_2_Cit and Na_3_Cit with Ca^2+^ ions.

pH	6.0	7.4	8.0
Et_2_Cit-CaCl_2_ system			
reaction order (*n*)	2.03	2.46	2.73
stability constants (*K*_*s*_)	10^0.93^	10^2.06^	10^3.06^
rate constant (*k*)/L·mol^−1^·s^−1^	9	120	4571
reverse reaction rate constant (*k*_re_)/L·mol^−1^·s^−1^	0.04	0.52	19.80
Na_3_Cit-CaCl_2_ system			
reaction order (*n*)	2.16	2.44	3.0
*K*_*s*_	10^1.98^	10^2.46^	10^4.83^
*k*/L·mol^−1^·s^−1^	60	289	13489
*k*_re_/L·mol^−1^·s^−1^	0.03	0.15	6.79

**Table 2:** Elemental analysis data and Ca content measured by the ICP of complex CaEt_2_Cit.

	C%	H%	Ca%
EA results	41.55 (41.64)*	5.73 (5.55)	—
ICP result	—	—	13.68 (13.93)

*The value in bracket was theoretical value, which is calculated according to the formula of complex CaEt_2_Cit.

**Table 3:** Wavenumber of the main absorption peaks of FT-IR spectra of Et_2_Cit and its complex CaEt_2_Cit.

Et_2_Cit/cm^−1^	3480 (OH)*	2986 (CH_2_, CH_3_)	1732 (O–C=O)	1100 (C–O–C)
CaEt_2_Cit/cm^−1^	3430 (OH)	2982 (CH_2_, –CH_3_)	1709, 1624 (O–C=O)	1081, 1041 (C–O–C)

**Table 4:** Absorption peak section and its assignment of the ^1^H NMR spectra of Et_2_Cit and CaEt_2_Cit.

Et_2_Cit/ppm	1.24~1.31 (–CH_3_)	2.80–2.97 (–CH_2_CO)	4.13~4.30 (–OCH_2_)	7.26, 6.28 (–OH)
CaEt_2_Cit/ppm	1.22~1.28 (–CH_3_)	2.77–2.98 (–CH_2_CO)	4.12~4.17 (–OCH_2_)	
